# Beweggründe von Patienten, die sich selbständig in der Notaufnahme vorstellen – eine prospektive monozentrische Beobachtungsstudie

**DOI:** 10.1007/s00063-024-01106-2

**Published:** 2024-01-17

**Authors:** Katharina Sitter, Mareen Braunstein, Markus Wörnle

**Affiliations:** 1 Klinik für Neurologie der Universität Regensburg am medbo Bezirksklinikum Regensburg, Universitätsstr. 84, 93053 Regensburg, Deutschland; 2 Zentrale Notaufnahme, Klinikum Innenstadt, LMU Klinikum, Ludwig-Maximilians-Universität München, Ziemssenstraße 5, 80336 München, Deutschland

**Keywords:** Fragebogen, Bildungsstand, Hausarzt, Selbsteinschätzung, Triage, Questionnaires, Education level, General practitioner, Self-assessment, Triage

## Abstract

**Hintergrund:**

In der aktuellen fachlichen, gesellschaftlichen und politischen Diskussion wird immer wieder die These aufgestellt, dass ein Großteil der Patienten, die selbständig die Notaufnahme aufsuchen, auch in anderen Versorgungsbereichen wie beim Hausarzt, dem kassenärztlichen Bereitschaftsdienst oder in Notfallpraxen behandelt werden könnte. Verschiedene Gründe werden aufgeführt, warum diese alternativen Versorgungsbereiche in diesen Fällen nicht genutzt werden.

**Ziel der Arbeit:**

In unserer Arbeit untersuchten wir die Beweggründe von Patienten, die sich selbständig in der Notaufnahme vorstellten, sowie soziodemografische Parameter dieses Studienkollektivs.

**Material und Methoden:**

Die Erhebung erfolgte im Rahmen einer prospektiven monozentrischen Beobachtungsstudie an internistischen Patienten einer universitären Notaufnahme in Innenstadtlage.

**Ergebnisse:**

1086 Patienten konnten in die Studie eingeschlossen werden. 33 % der Studienteilnehmer besuchten die Notaufnahme aufgrund einer ärztlichen Empfehlung bzw. Einweisung anstelle einer alternativen Versorgungsmöglichkeit. Der Hauptgrund für den Besuch der Notaufnahme war die subjektiv beurteilte Dringlichkeit der Beschwerden. 28 % der Patienten, die sich selbständig in der Notaufnahme vorstellten, mussten im Verlauf stationär weiterversorgt werden. Die Kenntnis von alternativen Versorgungswegen wie einer Inanspruchnahme des Rettungsdiensts, der Vorstellung beim kassenärztlichen Bereitschaftsdienst oder dem Besuch von Notfallpraxen war gering.

**Diskussion:**

Die Notaufnahmen sind nach wie vor eine wichtige Anlaufstelle für Patienten, die sich dort selbständig und ohne Einlieferung mit dem Rettungsdienst vorstellen. Die Beweggründe, warum die Patienten einen Besuch der Notaufnahme einer Behandlung in einer alternativen Versorgungsstruktur vorziehen, sind unterschiedlich. Wenn Alternativen anstatt Notaufnahmen genutzt werden sollen, so müssen hier erst Strukturen aufgebaut bzw. erweitert werden.

**Zusatzmaterial online:**

Zusätzliche Informationen sind in der Online-Version dieses Artikels (10.1007/s00063-024-01106-2) enthalten.

## Abstract (en)

**Background:**

In the ongoing professional, societal, and political discussion, the hypothesis is repeatedly put forward that a large portion of patients who independently visit the emergency department could also be treated in other care settings such as by a general practitioner, the statutory medical on-call service, or in emergency clinics. Various reasons are cited for why these alternative care settings are not utilized in these cases.

**Objectives:**

This study investigates the motives of patients who presented independently at the emergency department, as well as the socio-demographic parameters of this study cohort.

**Materials and methods:**

The survey was carried out as part of a prospective monocentric observational study of internal medicine patients at a university emergency department.

**Results:**

A total of 1086 patients were included in the study. In total, 33% of the study participants visited the emergency department based on a physician’s recommendation or referral instead of opting for an alternative care option. The main reason for visiting the emergency department was the subjectively assessed urgency of their symptoms. Among the patients who presented independently at the emergency department, 28% required further inpatient care during the course of treatment. Awareness of alternative care pathways, such as utilizing emergency medical services, seeking care from the statutory medical on-call service, or visiting an emergency clinic, was low.

**Conclusions:**

Emergency departments remain an important point of contact for patients who present there independently, without being brought by emergency medical services. The motives behind why patients choose a visit to the emergency department over treatment in an alternative care setting vary. If alternatives are to be used instead of emergency departments, structures first need to be established or expanded.

## Hintergrund und Fragestellung

Von vielen Kliniken wird zunehmend eine Fehlinanspruchnahme der Notaufnahmen durch Patienten beklagt, die eigentlich gar nicht in der Notaufnahme behandelt werden müssten. Warum diese Patienten die Notaufnahme der Behandlung durch alternative Versorgungsstrukturen vorziehen, wird in der aktuellen Literatur kontrovers diskutiert [13, 21, 24–26]. Denkbar sind die fehlende Vertrautheit mit den Strukturen des Gesundheitssystems, ein zunehmender Anspruch auf eine durchgehende Verfügbarkeit einer ärztlichen Versorgung, zu lange Wartezeiten auf Termine in den Arztpraxen, veränderte Altersstrukturen der Bevölkerung verbunden mit veränderten Komorbiditäten oder eine unzureichende interdisziplinäre Gesundheitsversorgung im ambulanten Bereich [24].

Als Reaktion auf diese Situation sind vonseiten der Politik grundlegende Maßnahmen zur Reform der Notfallversorgung geplant [10].

Ziel unserer Studie war es, die Beweggründe von Patienten zu erfragen, die sich selbständig in unserer Notaufnahme vorstellten und eine Behandlung in der Notaufnahme einer alternativen Versorgung vorzogen. Neben subjektiven Kriterien sollten auch objektive soziodemografische Faktoren in unserem Studienkollektiv erfasst werden. Als Hypothese wurde angenommen, dass ein Teil der Patienten, die sich selbständig in der Notaufnahme vorstellten, auch in alternativen Versorgungsbereichen behandelt werden könnte.

## Studiendesign und Untersuchungsmethoden

Die Erhebung erfolgte im Rahmen einer deskriptiven, prospektiven monozentrischen Beobachtungsstudie vom 1. Mai 2018 bis 31. August 2019 in der interdisziplinären Notaufnahme des Klinikums der Ludwig-Maximilians-Universität München (LMU) am Campus Innenstadt. Dafür wurde internistisch triagierten Patienten, die sich selbständig in der Notaufnahme vorstellten, ein Fragebogen mit vorgegebenen Antwortkategorien ausgehändigt (Abb. Suppl. 1). Zum Studienzeitpunkt erfolgte die Behandlung für internistische und chirurgische Patienten in der Notaufnahme am Campus Innenstadt noch in getrennten räumlichen Bereichen mit jeweils eigenem Behandlungstresen. Die Fächerzuteilung erfolgte vor Behandlung an den Behandlungstresen. Der Fragebogen beinhaltete Fragen zum soziodemografischen Hintergrund, zu aktuellen Beschwerden, zu Fragen zur hausärztlichen Versorgung sowie zum Bekanntheitsgrad alternativer Anlaufstellen. Die Einteilung in drei verschiedene Bildungskategorien (hoch, mittel, niedrig) erfolgte anhand der Bildungsklassifikation Comparative Analysis of Social Mobility in Industrial Nations (CASMIN; [2]). Zusätzlich sollten die Patienten ihre Behandlungsdringlichkeit selbst einschätzen und Gründe für das Aufsuchen der Notaufnahme angeben. Teilweise waren Mehrfachantworten möglich. Eingeschlossen wurden erwachsene Patienten. Ein Ausschluss von der Studie erfolgte, wenn eine verbale Verständigung auf Deutsch nicht möglich war, bei fehlender Einwilligungsfähigkeit, bei schwerwiegenden Einschränkungen des Sehvermögens, bei schweren funktionellen Einschränkungen, bei Analphabetismus, bei Minderjährigkeit und bei Einweisung durch den Rettungsdienst.

Ab dem 1. Januar 2019 erfolgte eine standardisierte Ersteinschätzung im Rahmen der 5‑stufigen ESI (Emergency Severity Index)-Triage. Die Triage wurde von geschultem Pflegepersonal durchgeführt. Die Triage und Dokumentation erfolgte mit der epias-Notaufnahmesoftware (epias GmbH, 65510 Idstein, Deutschland).

Im Fragebogen zur subjektiven Selbsteinschätzung der Dringlichkeit der Beschwerden wurde seit Beginn der Studie ein 3‑stufiges System verwendet, das auch nach dem 1. Januar 2019 nicht verändert wurde.

Die Zustimmung der Ethikkommission der Medizinischen Fakultät der LMU München wurde im April 2019 erteilt (18-201). Die Studie wurde im Deutschen Register Klinischer Studien (DRKS00033059) eingetragen. Die Patienten wurden über das Ziel der Studie durch einen zusätzlichen Informationsbogen aufgeklärt. Aufgrund des deskriptiven Charakters der Studie wurde keine statistische Auswertung durchgeführt. Die wissenschaftliche Erhebung und Präsentation der Daten orientierte sich an der „reporting guideline“ für Querschnittsstudien [35].

Aus Gründen der besseren Lesbarkeit wird auf die gleichzeitige Verwendung der Sprachformen männlich, weiblich und divers (m, w, d) verzichtet. Sämtliche Personenbezeichnungen gelten gleichermaßen für alle Geschlechter. In unserer Institution gibt es keine verbindlichen Vorgaben zur sprachlichen Gleichbehandlung.

## Ergebnisse

### Patientenzahlen im Beobachtungszeitraum

Im Beobachtungszeitraum wurden insgesamt 12.071 Patienten in der internistischen Notaufnahme des Klinikums Innenstadt der LMU behandelt. Die Anzahl der Patienten, die sich selbständig vorstellten (*N* = 6122 [51 %]), war vergleichbar mit der Zahl der Patienten, die durch den Rettungsdienst eingewiesen wurden (*n* = 5949 [49 %]). Von den 6122 Patienten, die sich selbständig vorstellten, nahmen 1086 (18 %) an der Umfrage teil.

### Soziodemografische Charakterisierung des Studienkollektivs

**Abb. 1:**
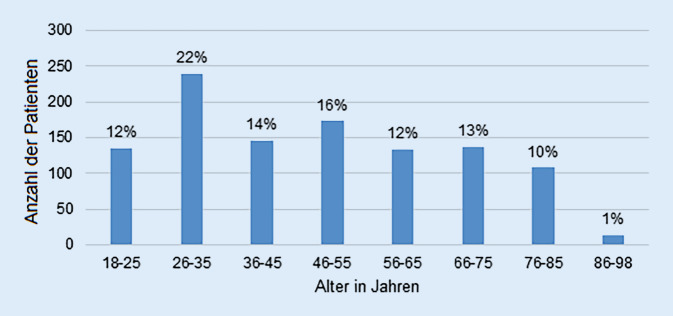
Altersstruktur des Studienkollektivs

Die Geschlechterverteilung in der Studienpopulation war mit 515 Frauen und 571 Männern ausgewogen. Die Altersverteilung der Patienten ist in Abb. 1 dargestellt. 496 Patienten waren ledig, 424 verheiratet, 91 geschieden und 74 verwitwet. 71 % der Patienten hatten die deutsche Staatsbürgerschaft, 21 % eine europäische und 8 % eine andere Staatsbürgerschaft. Basierend auf ihrer schulischen und universitären Ausbildung wurden die Studienteilnehmer in drei Bildungskategorien eingeteilt: 1. Fachhochschul- oder Universitätsabschluss (hoch), 2. Realschulabschluss oder Abitur (mittel), 3. kein Schulabschluss oder Hauptschulabschluss (niedrig). 41 % der Studienteilnehmer besaßen Abitur oder Realschulabschluss (mittlere Bildungskategorie), 32 % hatten einen Fachhochschul- oder Hochschulabschluss (hohe Bildungskategorie) und 27 % hatten einen Hauptschulabschluss oder gar keinen Schulabschluss (niedrige Bildungskategorie). 44 % der Patienten waren als Angestellte tätig. 25 % waren Rentner, 10 % selbständig, 10 % Schüler, Studenten oder Auszubildende. 4 % waren Hausfrau/-mann, 3 % arbeitssuchend, 2 % Beamte und 3 % fielen unter Sonstige. 935 Patienten (86 %) waren gesetzlich versichert, 137 Patienten (13 %) waren privat versichert und 12 Patienten (1 %) gaben an, keine Versicherung zu haben.

### Ärztliche Betreuung vor der Vorstellung in der Notaufnahme

**Abb. 2:**
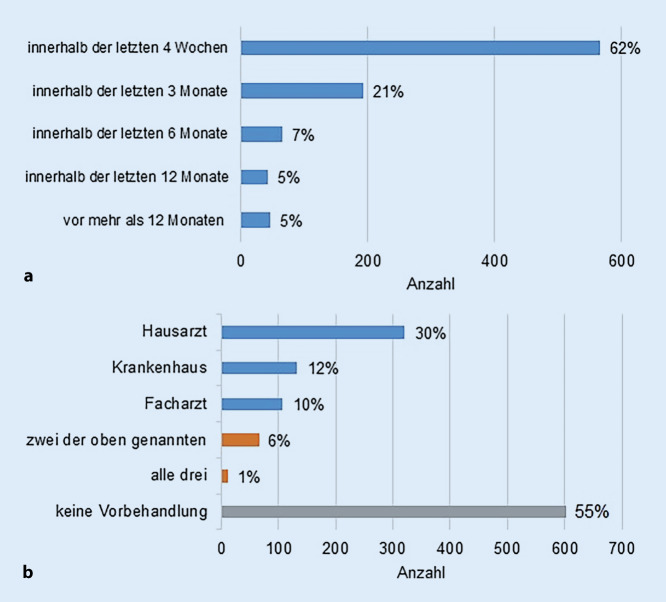
**a** Zeitpunkt des letzten hausärztlichen Besuchs der Patienten (keine Angabe = 39), **b** Vorbehandlungen aufgrund der aktuellen Beschwerden in den letzten vier Wochen (keine Angabe = 14): Mehrfachantworten möglich

950 Studienteilnehmer (88 %) befanden sich in regelmäßiger hausärztlicher Betreuung. Davon beantworteten 911 Patienten die Frage nach dem Zeitpunkt des letzten Hausarztbesuchs. 62 % dieser Patienten suchten ihren Hausarzt innerhalb der letzten vier Wochen auf. 21 % wurden in den letzten drei Monaten von ihrem Hausarzt behandelt und bei insgesamt 17 % lag der letzte Hausarztbesuch länger als drei Monate zurück (Abb. 2a).

Es wurde genauer untersucht, inwiefern die Patienten aufgrund ihrer aktuellen Beschwerden bereits eine ärztliche Vorbehandlung in Anspruch genommen hatten. 55 % hatten diesbezüglich keine Vorbehandlung. 45 % der Befragten gaben an, dass Sie innerhalb der letzten vier Wochen aufgrund ihrer derzeitigen Beschwerden ärztlich behandelt worden waren. Insgesamt waren 30 % vor dem Aufsuchen der Notaufnahme bereits bei ihrem Hausarzt, 12 % im Krankenhaus und 10 % bei einem Facharzt. 6 % hatten zwei der drei Institutionen aufgesucht und 1 % alle drei. Mehrfachantworten waren möglich (Abb. 2b). 151 Patienten (14 %) kamen mit einer schriftlichen Einweisung durch den Hausarzt, einen Facharzt oder eine kassenärztliche Bereitschaftspraxis in die Notaufnahme. 935 Studienteilnehmer (86 %) suchten die Notaufnahme ohne schriftliche ärztliche Zuweisung auf.

### Kenntnis alternativer Anlaufstellen der Notfallversorgung

**Abb. 3:**
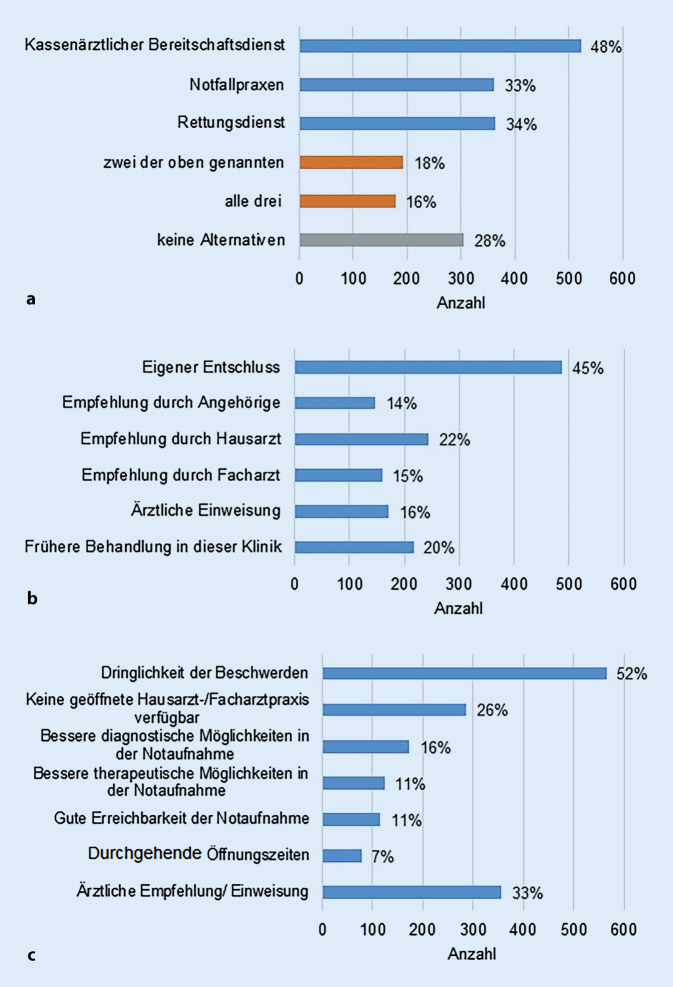
**a** Bekanntheit alternativer Anlaufstellen zur Notfallversorgung im Studienkollektiv (keine Angabe = 81), Mehrfachantworten möglich, **b** Empfehlung zum Aufsuchen der Notaufnahme (keine Angabe = 13), Mehrfachantworten möglich, **c** Gründe für den Besuch der Notaufnahme und gegen den Besuch eines Haus- oder Facharztes (keine Angabe = 36), Mehrfachantworten möglich

Bei der Frage nach der Kenntnis alternativer Anlaufstellen der Notfallversorgung standen der Bereitschaftsdienst der kassenärztlichen Vereinigung, die Notfallpraxen sowie der Rettungsdienst als Antworten zur Auswahl. Mehrfachantworten waren auch hier möglich. 48 % der Studienteilnehmer kannten den kassenärztlichen Bereitschaftsdienst, 33 % die Notfallpraxen und 34 % den Rettungsdienst. Nur 18 % hatten Kenntnis von allen drei genannten Alternativen. 28 % der Patienten waren die Alternativen nicht bekannt (Abb. 3a).

### Gründe für das Aufsuchen der Notaufnahme

Es wurde auch erfragt, auf welche Empfehlung hin die Patienten die Notaufnahme aufsuchten. 45 % der gesamten Studienteilnehmer suchten die Notaufnahme aus eigenem Entschluss auf. 22 % bzw. 15 % kamen in die Notaufnahme, da Ihnen ihr Hausarzt bzw. ein Facharzt dazu geraten hatte. Bei 16 % erfolgte eine schriftliche stationäre Einweisung durch einen Arzt. 14 % wurde der Besuch der Notaufnahme durch Angehörige empfohlen. 20 % der Patienten kamen aufgrund einer früheren Behandlung in derselben Klinik in die Notaufnahme (Abb. 3b). Im Fragebogen wurde auch erfragt, warum sich die Patienten für den Besuch der Notaufnahme und gegen den Besuch eines Haus- oder Facharztes entschieden hatten. Als häufigster Grund für das Aufsuchen der Notaufnahme wurde die Dringlichkeit der Beschwerden (52 %) angegeben. 26 % suchten die Notaufnahme auf, da keine geöffnete Hausarzt- oder Facharztpraxis verfügbar war. Bessere diagnostische Möglichkeiten erwarteten 16 % der Patienten und bessere therapeutische Möglichkeiten 11 %. Wegen der guten Erreichbarkeit der Notaufnahme kamen 11 % in die Notaufnahme und die durchgehende Öffnungszeit nutzten 7 % der Patienten (Abb. 3c).

**Abb. 4:**
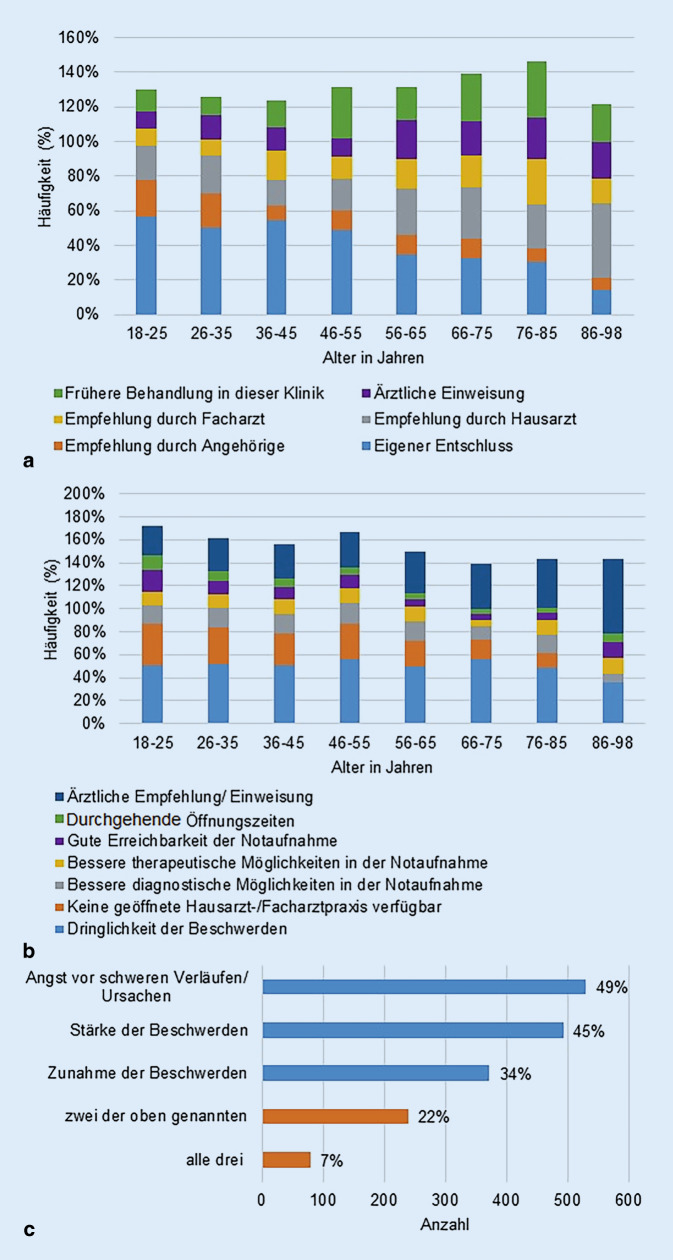
**a** Empfehlung zum Aufsuchen der Notaufnahme in Abhängigkeit vom Alter (Mehrfachantworten möglich), **b** Gründe für den Besuch der Notaufnahme und gegen den Besuch eines Haus- oder Facharztes in Abhängigkeit vom Alter (Mehrfachantworten möglich), **c** Begründung für die individuelle Selbsteinschätzung der Dringlichkeit der Beschwerden (keine Angabe = 90), Mehrfachantworten möglich

Die Empfehlungen zum Besuch der Notaufnahme waren altersabhängig unterschiedlich. Während jüngere Patienten vorwiegend aus eigenem Entschluss die Notaufnahme aufsuchten, waren mit zunehmendem Alter die Hauptgründe eine Empfehlung durch einen Haus- bzw. Facharzt sowie eine stationäre ärztliche Einweisung (Abb. 4a). Auch bei der Begründung für das Aufsuchen der Notaufnahme und gegen einen Besuch beim Haus- oder Facharzt zeigten sich altersabhängige Unterschiede. Vor allem ältere Studienteilnehmer suchten auch hier die Notaufnahme auf ärztliche Empfehlung oder mit einer stationären ärztlichen Einweisung auf (Abb. 4b).

### Subjektive Selbsteinschätzung der Beschwerden

Die Patienten wurden zusätzlich befragt, wie sie die Dringlichkeit ihrer Beschwerden selbst einschätzten. Dabei stuften 52 % der Studienteilnehmer ihre Beschwerden als dringlich, 42 % als sehr dringlich und 6 % als eher nicht dringlich ein. Geschlecht sowie Bildungsstand hatten keinen Einfluss auf die subjektive Einschätzung der Dringlichkeit der Beschwerden. Allerdings wurden die Beschwerden mit zunehmendem Alter als dringlicher eingeschätzt.

Auf die Frage nach der Begründung für die individuelle Selbsteinschätzung der Dringlichkeit wurden drei Antwortmöglichkeiten zur Auswahl gestellt, wobei wieder Mehrfachantworten möglich waren. 90 Patienten gaben hierzu keine Angabe. 49 % der verbliebenen Studienteilnehmer gaben die Angst vor schweren Verläufen oder Ursachen an, 45 % nannten die Stärke der Beschwerden und 34 % die Zunahme der Beschwerden als Begründung. Von 22 % der Patienten wurden zwei Gründe angegeben, von 7 % der Studienteilnehmer wurden alle drei Gründe angegeben (Abb. 4c). In der Gruppe der Patienten, die ihre Behandlungsdringlichkeit am höchsten einschätzten wurde die Behandlungsdringlichkeit am häufigsten mit der Stärke der Beschwerden begründet (45 %). Bei Patienten, die ihre Behandlungsdringlichkeit selbst als niedrig einstuften, war hingegen die Angst vor schweren Verläufen oder Ursachen der häufigste Grund (52 %).

### Weiterer Verbleib der Studienteilnehmer nach der Behandlung in der Notaufnahme

**Abb. 5:**
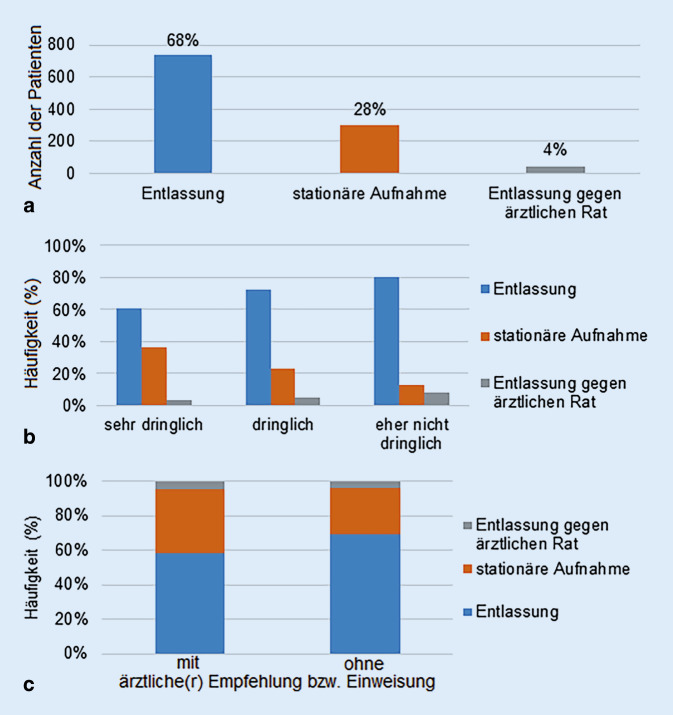
**a** Weiterer Verbleib der Patienten nach der Behandlung in der Notaufnahme, **b** weiterer Verbleib der Patienten nach der Behandlung in der Notaufnahme in Abhängigkeit von der individuellen Selbsteinschätzung der Dringlichkeit der Beschwerden, **c** Weiterer Verbleib der Patienten nach der Behandlung in der Notaufnahme in Abhängigkeit von der Zuweisungsart

Es wurde auch erfasst, ob die Patienten nach ihrer Behandlung in der Notaufnahme wieder entlassen werden konnten oder ob eine stationäre Weiterversorgung notwendig war. 68 % der Studienteilnehmer konnten direkt aus der Notaufnahme wieder entlassen werden, während 28 % der Patienten stationär aufgenommen werden mussten. 4 % der Patienten haben die Klinik gegen ärztlichen Rat verlassen (Abb. 5a).

Danach wurde überprüft, ob die subjektive Selbstbeurteilung der Dringlichkeit der Beschwerden einen Einfluss darauf hatte, ob eine Entlassung nach der Behandlung in der Notaufnahme möglich war oder ob eine stationäre Weiterversorgung erfolgen musste. 452 Studienteilnehmer (42 %) schätzten ihre Beschwerden selbst als sehr dringlich ein. Von dieser Patientengruppe konnten im Verlauf 61 % entlassen werden, 36 % mussten stationär aufgenommen werden und 3 % verließen die Notaufnahme gegen ärztlichen Rat. 555 Studienteilnehmer (52 %) gaben an, dass ihre Beschwerden dringlich seien. Von diesen Patienten wurden 72 % entlassen, 23 % stationär aufgenommen und 5 % gegen ärztlichen Rat entlassen. Von den 65 Studienteilnehmern (6 %), die ihre Beschwerden selbst als nicht dringlich einschätzten, wurden 80 % wieder entlassen, 12 % zur stationären Weiterversorgung aufgenommen und 8 % gegen ärztlichen Rat entlassen (Abb. 5b).

In unserer Befragung gaben 22 % der Patienten an, dass sie die Notaufnahme auf Empfehlung ihres Hausarztes aufsuchten, 15 % kamen auf Empfehlung eines Facharztes und 16 % hatten eine schriftliche ärztliche Einweisung, wobei hier auch Mehrfachantworten möglich waren (Abb. 3b). Der weitere Verbleib der Patienten, die aufgrund einer ärztlichen Empfehlung oder schriftlichen Einweisung in die Notaufnahme kamen, unterschied sich von dem der Studienteilnehmer, die ohne ärztliche Empfehlung kamen. 37 % der Studienteilnehmer, die aufgrund einer ärztlichen Empfehlung bzw. Einweisung die Notaufnahme aufsuchten, wurden stationär weiterversorgt. In der Gruppe der Patienten ohne ärztliche Empfehlung oder Einweisung wurden 26 % stationär aufgenommen (Abb. 5c).

### Dauer und Bereich der Beschwerden

Im Folgenden wurde die Dauer der Beschwerden erfragt, die zur aktuellen Vorstellung in der Notaufnahme führten. Bei 26 % der Studienteilnehmer traten die Beschwerden innerhalb der letzten 12 h, bei 23 % innerhalb von 12 bis 48 h auf. Bei 32 % der Patienten bestanden die Beschwerden seit Tagen, bei 13 % seit Wochen und bei 6 % seit Monaten.

Die Patienten sollten auch selbst einschätzen, in welchem Organsystem ihre aktuellen Beschwerden oder Schmerzen lagen. Hier wurden im Fragebogen acht Antwortmöglichkeiten vorgegeben, wobei auch wieder Mehrfachantworten möglich waren. 33 % lokalisierten ihre Beschwerden im Herz-Kreislauf-System, 27 % im Verdauungssystem bzw. Bauch, 23 % in den Atmungsorganen, 18 % im Kopf-Hals-Bereich, 17 % im Bewegungsapparat bzw. den Gelenken, 12 % im Rücken und 6 % im Hautbereich. Einen allgemeinen Bereich bzw. Fieber gaben 15 % als Antwort an.

### Behandlungsdiagnosen in der Notaufnahme

**Abb. 6:**
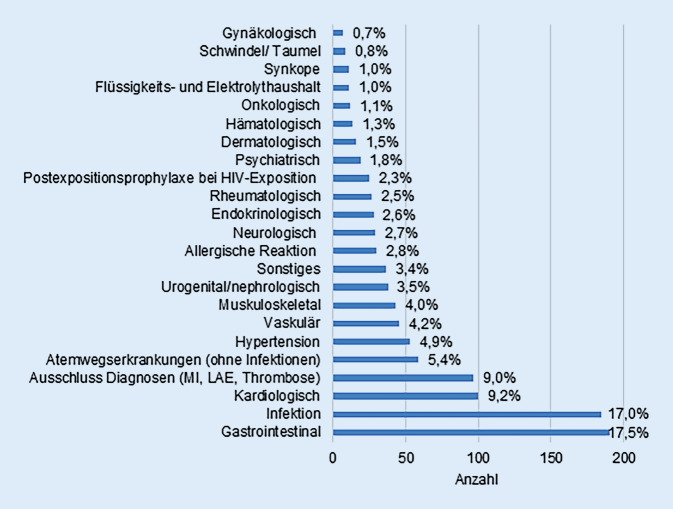
Diagnosen im Studienkollektiv. *MI* Myokardinfarkt, *LAE* Lungenarterienembolie

Die in der Notaufnahme gestellten Diagnosen wurden für die Patienten, die an der Studie teilnahmen in Diagnosegruppen zusammengefasst (Abb. 6). Die Zusammenfassung orientierte sich dabei an den Kapiteln bzw. Gruppen der ICD-10-GM-Version 2019. Die häufigsten Diagnosegruppen waren gastrointestinale Erkrankungen (17,5 %), Infektionen (17 %) und kardiologische Erkrankungen (9,2 %). Die Ausschlussdiagnosen wurden in einer eigenen Gruppe zusammengefasst, da bei vielen symptomatischen Patienten ein Myokardinfarkt, eine Lungenarterienembolie oder eine Thrombose ausgeschlossen werden musste. Die drei größten Diagnosegruppen wurden näher untersucht. Bei den gastroenterologischen Erkrankungen (*n* = 190) waren allgemeine gastrointestinale Beschwerden mit Übelkeit, Erbrechen und Diarrhö führend (52,7 %). Bei den Infektionen (*n* = 185) kam es am häufigsten zu Harnwegsinfekten (16,8 %), Infektionen der oberen Atemwege (16,2 %) und Pneumonien (15,1 %). Innerhalb der kardiologischen Erkrankungen (*n* = 100) waren die am häufigsten gestellten Diagnosen Palpitation (20 %), Herzinsuffizienz (19 %) und Vorhofflimmern (17 %).

### Strukturierte Ersteinschätzung der Patienten

**Abb. 7:**
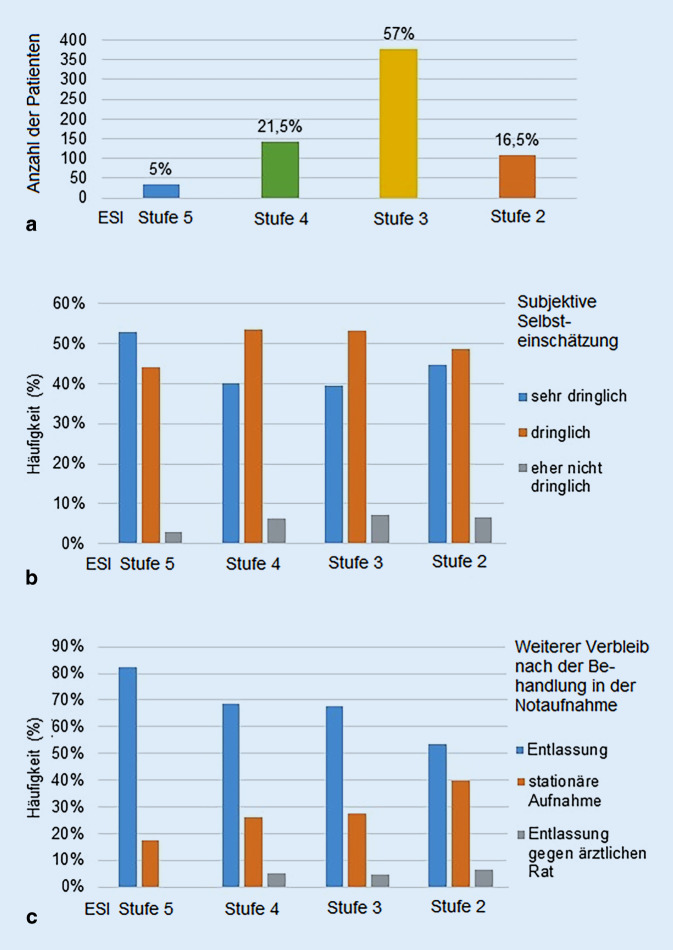
**a** Einteilung der Patienten nach Behandlungsdringlichkeit mittels Emergency-Severity-Index(ESI)-Triage, **b** individuelle Selbsteinschätzung der Dringlichkeit der Beschwerden und objektive Beurteilung der Behandlungsdringlichkeit mittels ESI-Triage (keine Angabe = 9), **c** weiterer Verbleib der Patienten nach der Behandlung in der Notaufnahme in Abhängigkeit von ESI-Triage

Seit dem 1. Januar 2019 erfolgte in unserer Notaufnahme die strukturierte Ersteinschätzung durch Bestimmung des Emergency Severity Index (ESI). Dabei handelt sich um eine Beurteilung der Behandlungsdringlichkeit durch einen 5‑stufigen standardisierten Algorithmus. Je niedriger die Stufe, desto dringlicher wird die Notfallbehandlung beurteilt. In Abb. 7a ist die Verteilung der 660 Patienten dargestellt, bei denen eine Triage mittels ESI durchgeführt wurde und die in die Studie eingeschlossen werden konnten. 5 % der Patienten wurden der Stufe 5 mit sehr niedriger Behandlungsdringlichkeit zugeteilt, 21,5 % der Stufe 4, 57 % der Stufe 3 und 16,5 % der Stufe 2. Im Beobachtungszeitrum konnte kein Patient, der sich selbständig vorstellte und der Stufe 1 mit der höchsten Behandlungsdringlichkeit hätte zugeteilt werden müssen, in die Studie eingeschlossen werden. Im Zeitraum vom 1. Januar bis 31. August 2019 wurden nur fünf Patienten, die sich selbständig vorstellten, in die Stufe 1 triagiert. Da seit Beginn der Studie die subjektive Selbsteinschätzung der Dringlichkeit der Beschwerden durch die Patienten in einer 3‑stufigen Beurteilung erfolgte, wurde dieser Teil des Fragebogens auch nach dem 1. Januar 2019 so weitergeführt. Eine Anpassung an die 5‑stufige ESI-Einschätzung erfolgte nicht. Es wurde überprüft, ob die subjektive Selbsteinschätzung der Patienten mit der strukturierten Ersteinschätzung mittels ESI in der Notaufnahme übereinstimmen würde. In der in der Studie mit der dringlichsten Behandlungspriorität erfassten Stufe 2 schätzten 44 % der Studienteilnehmer ihre Behandlungsdringlichkeit als sehr dringlich ein. In der objektiv am wenigsten dringlichen Priorisierungsstufe 5 schätzten 53 % der Patienten ihre Behandlungsdringlichkeit als sehr hoch ein (Abb. 7b). Daneben wurde untersucht, ob die Einteilung der Studienteilnehmer in die Triagestufen einen Einfluss auf den weiteren Verbleib nach der Behandlung in der Notaufnahme hatte. Der Anteil der Patienten, die stationär weiterversorgt werden mussten, nahm zu, je dringlicher die Behandlung eingeschätzt wurde. Während in der 5. Stufe nur 18 % der Patienten stationär aufgenommen werden mussten, wurden in der 2. Stufe 40 % der Patienten stationär weiterversorgt (Abb. 7c).

## Diskussion

In aktuellen Publikationen werden verschiedene Gründe diskutiert, weshalb in den letzten Jahren immer mehr Patienten die Notaufnahmen in Anspruch nehmen [31, 32]. Dabei beschreiben mehrere Studien, dass zunehmend Patienten, die nicht die Kriterien eines medizinischen Notfalls erfüllen, mit nichtdringlichen Behandlungsanlässen die Notaufnahmen aufsuchen. Die Zahlenangaben, die in der Literatur beschrieben werden, variieren aber erheblich. In mehreren Arbeiten wird die Zahl der Patienten, die die Notaufnahme mit nichtdringlichen Beschwerden aufsuchen, mit über 30 % angegeben, andere Studien gehen von Werten unter 10 % aus [4, 7, 9, 12, 13, 16, 19, 20, 29].

Wir haben in unserer Arbeit näher untersucht, warum sich Patienten selbständig in unserer Notaufnahme vorstellten, anstatt andere Versorgungsstrukturen in Anspruch zu nehmen.

In unserer Studienpopulation gaben 33 % der Patienten an, dass sie aufgrund einer ärztlichen Empfehlung die Notaufnahme aufsuchten anstelle eines Besuchs beim niedergelassenen Haus- oder Facharzt. Eine dokumentierte haus- oder fachärztliche Einweisung lag allerdings nur bei 16 % der Patienten vor. Hier gab es altersbedingte Unterschiede. Vor allem ältere Patienten kamen auf Empfehlung bzw. Einweisung durch einen Arzt in die Notaufnahme, wohingegen für jüngere Patienten strukturelle und organisatorische Gründe im Vordergrund standen. Diese Beobachtungen konnten in verschiedenen anderen internationalen Studien zum Teil nachvollzogen werden [34]. Der überwiegende Anteil der Studienteilnehmer kam aus eigenem Entschluss in die Notaufnahme. Bemerkenswert ist auch, dass 88 % der Studienteilnehmer angaben, sich in regelmäßiger hausärztlicher Betreuung zu befinden. 45 % der Studienteilnehmer waren sogar wegen ihrer aktuellen Beschwerden bereits in einem Krankenhaus oder bei einem niedergelassenen Arzt vorbehandelt worden.

Eine Besonderheit unserer Notaufnahme ist, dass sich in ca. einem Kilometer Entfernung die ärztliche Bereitschaftspraxis im Elisenhof befindet. Diese Praxis ist nicht organisatorisch mit unserer Klinik verknüpft, zählt aber zu den größten Bereitschaftspraxen in Deutschland und hat täglich bis in die späten Abendstunden geöffnet.

Die Frage, wie viele der Patienten unseres Studienkollektivs auch in alternativen Versorgungsbereichen, wie z. B. beim Hausarzt oder in einer Bereitschaftspraxis, hätten behandelt werden können, ist nicht leicht zu beantworten. Es handelte sich um Patienten, die in der Lage waren, sich eigenständig fußläufig in der Notaufnahme vorzustellen und vor Behandlungsbeginn einen Fragebogen auszufüllen. Diese Tatsache liefert zwar erste Hinweise dafür, dass das Vorliegen einer akuten lebensbedrohlichen Erkrankung unwahrscheinlich ist, schließt aber eine hohe Behandlungsdringlichkeit nicht von vorherein aus. Immerhin mussten 28 % der Patienten stationär weiterbehandelt werden. Auch der Ausschluss einer lebensbedrohlichen Erkrankung benötigt teilweise apparative Diagnostik, die in einer Bereitschaftspraxis nicht rund um die Uhr zur Verfügung steht. In modernen Notaufnahmen erfolgen mittlerweile regelmäßig viele diagnostische und therapeutische Maßnahmen innerhalb kürzester Zeit, die eigentlich weit über das Maß einer einfachen ambulanten Behandlung hinausgehen. Die Tatsache, dass die Patienten wieder aus der Notaufnahme entlassen wurden, bedeutet nicht, dass die Notfalldiagnostik und -therapie, die in der Notaufnahme durchgeführt wurde, ohne Weiteres auch durch eine Bereitschaftspraxis erbracht werden könnte.

Ein wichtiger Faktor ist sicher die eigene Selbstwahrnehmung der Patienten. Nach Etablierung eines 5‑stufigen Triagealgorithmus (ESI-Triagesystem) in der Notaufnahme im Januar 2019 bot sich die Möglichkeit, bei einer Subgruppe von 660 Patienten die objektive Behandlungsdringlichkeit mit der subjektiven Selbstwahrnehmung zu vergleichen. Dabei zeigte sich eine deutliche Diskrepanz. Von den im Triagealgorithmus als Stufe 2 kategorisierten Patienten schätzten lediglich 44 % ihre Beschwerden als sehr dringlich ein, wohingegen dieser Anteil bei den der niedrigsten Behandlungspriorität zugeteilten Patienten bei 53 % lag. Die Zahl der Patienten, die ihre eigene Behandlungsdringlichkeit als eher nicht dringlich einschätzten, lag durchweg unter 10 %. Das Geschlecht, das Alter sowie der Bildungsstand hatten dabei keinen Einfluss auf die Selbstwahrnehmung der Dringlichkeit. Daneben zeigte sich eine sehr gute Korrelation der strukturierten Ersteinschätzung mit dem weiteren Verbleib der Patienten nach der Behandlung in der Notaufnahme. Je dringlicher die Beschwerden eingeschätzt wurden, desto höher war die stationäre Aufnahmequote. Die teilweise von der Einschätzung der Triage bzw. der Behandler abweichende Selbstwahrnehmung der Patienten ist dadurch erklärbar, dass es sich dabei um ein stark subjektiv beeinflusstes Kriterium handelt. In der Vergangenheit wurden deshalb auch verschiedene Schulungsprogramm für Patienten entwickelt. Die Einführung sogenannter Awareness-Programme, die den Patienten anhand von Informationsmaterial erklären sollten, in welchen Fällen ein Besuch in der Notaufnahme indiziert sei, hatte allerdings keinen signifikanten Effekt auf die Zahl der Notaufnahmebesuche [22]. Aber auch Schulungsprogramme für spezifischere Erkrankungen wie Diabetes mellitus und Asthma bronchiale oder Intensivprogramme für geriatrische oder chronisch kranke Patienten erbrachten widersprüchliche Ergebnisse [1, 3, 23, 26–28].

Ein weiteres Problem scheint zu sein, dass vielen Patienten Alternativen zur Notfallversorgung nicht bekannt sind. Dies hat sich auch in unserer Studie gezeigt. Nur 48 % der Studienteilnehmer kannten den kassenärztlichen Bereitschaftsdienst, 33 % die Notfallpraxen und 34 % den Rettungsdienst. 28 % der Patienten kannten keine der genannten Alternativen. Wichtig wäre es, sowohl den Bekanntheitsgrad der ambulanten Versorgungsstrukturen zu fördern als auch die Popularität der Telefonnummer 116117 des ärztlichen Bereitschaftsdiensts der Kassenärztlichen Vereinigungen gezielt zu steigern. Es ist allerdings nicht klar, ob eine telefonische Konsultation für Patienten hinsichtlich ihrer aktuellen Beschwerden wirklich zu einem Rückgang der Besuche der Notaufnahme führt. In Großbritannien führte die Einführung eines fachlich besetzten telefonischen Beratungsdiensts nicht zu einem Rückgang der Notaufnahmebesuche [17, 32, 33].

Unsere Studie ist die bislang größte Erhebung, die in dieser Form in Deutschland durchgeführt wurde. Eine mit 1175 Patienten ähnlich große Zahl an Studienteilnehmern wurde in eine Studie eingeschlossen, die in fünf Zentren in Hamburg und in Schleswig-Holstein parallel durchgeführt wurde [24]. Allerdings erfolgte hier keine kontinuierliche Datenerhebung über einen längeren Zeitraum. Die Tage, die zur Analyse herangezogen wurden, wurden für die jeweiligen Zentren zufällig ausgewählt, sodass die Gesamtzahl der Tage jeweils einem Äquivalent von zwei vollständigen Arbeitswochen entsprach. Außerdem wurden automatisch Patienten, die im Manchester Triage System als sofort behandlungspflichtig (rot) oder sehr dringend (orange) beurteilt wurden, ausgeschlossen. Einige Patienten mit niedriger Behandlungsdringlichkeit wurden ebenfalls nicht erfasst, da bei freien Notaufnahmekapazitäten eine Behandlung erfolgte, noch bevor der Fragebogen verteilt werden konnte. Unsere Erhebung erstreckte sich kontinuierlich über einen Zeitraum von 16 Monaten. Jahreszeitabhängige Variablen wie Monate mit einer hohen Zahl an viralen Atemwegsinfektionen oder Urlaubszeiten konnten so relativiert werden. In unserer Studie gab es auch keinen Ausschluss von Patienten mit besonders dringlicher Behandlungsindikation, auch wenn im Untersuchungszeitraum kein Studienteilnehmer in Stufe 1 (rot) triagiert wurde. Andere Studien mit ähnlicher Fragestellung berufen sich auf deutlich geringere Fallzahlen [6].

Unsere Studie zeigt sicher einige Schwächen. Die Rücklaufquote der Fragebögen war mit 18 % gering. Weshalb nur 1086 verwertbare Fragebögen für die Analyse zur Verfügung standen, kann nur vermutet werden. Der Fragebogen sollte allen für die Studie infrage kommenden Patienten durch das Pflegepersonal der Notaufnahme ausgehändigt werden. Dieser Weg bringt methodisch das Risiko mit sich, dass bei hohem Patientenaufkommen und dadurch entstehenden Spitzenbelastungen für das Personal die Verteilung des Fragebogens möglicherweise zurückgestellt wird. Auch ist nicht klar, wie viele der ausgehändigten Fragebögen wirklich ausgefüllt wurden. Die Beantwortung der Fragen war ausschließlich in schriftlicher Form durch die Patienten selbst möglich. Bei Unklarheiten waren keine Nachfragen möglich. Dies könnte dazu geführt haben, dass Patienten den Erhebungsbogen nicht verwertbar ausgefüllt haben. Dennoch wurde ein Fragebogen anstelle eines persönlichen Interviews zur Datenerhebung bevorzugt, da wir davon ausgingen, dass die Antworten so ehrlicher ausfallen würden. Auch glauben wir, dass durch ein persönliches Interview eine Selektion der Teilnehmer dahingehend erfolgt wäre, dass scheue und weniger motivierte Patienten nicht an der Umfrage teilgenommen hätten. Die ständige Verfügbarkeit eines persönlichen Interviewers über einen so langen Beobachtungszeitraum wäre auch aufgrund der begrenzten personellen Ressourcen nicht umsetzbar gewesen. Da der Fragebogen nur in deutscher Sprache vorlag, sind Migranten vermutlich unterrepräsentiert. Allerdings hatten doch 29 % der Studienteilnehmer eine andere als die deutsche Staatsbürgerschaft.

Rückblickend sind einige Verbesserungen möglich. Zum einen sollte den Patienten ein Nachfragen ermöglicht werden, was allerdings mit einem deutlich höheren personellen Aufwand verbunden ist. Zum anderen sollten die Fragebögen mehrsprachig vorliegen, um auch nicht deutschsprachige Patienten in die Studie einschließen zu können. Durch festgelegte Antwortmöglichkeiten wurde das Risiko einer subjektiven Interpretation der Antworten bei der Auswertung der Daten minimiert. Auch wenn Fragen mit vorgegebenen Antwortmöglichkeiten die Auswertung und Vergleiche erleichtern, könnten offene Fragen noch mehr persönliche Gründe von Patienten widerspiegeln. Da die Studie ausschließlich in der internistischen Notaufnahme durchgeführt wurde, sind die Krankheitsbilder bis auf wenige Ausnahmen auch diesem Fachbereich zuzuschreiben. Eine Übertragung auf andere Fachbereiche ist wahrscheinlich nicht automatisch möglich. In unserer jetzigen zentralen Notaufnahme könnten auch Patienten anderer Fachrichtungen eingeschlossen werden.

Die methodischen Probleme, die bei der wissenschaftlichen Aufarbeitung der Fragestellung, warum sich Patienten selbständig in der Notaufnahme vorstellen, auftreten, zeigen sich auch beim Vergleich der bestehenden Literatur zu diesem Thema. Zum einen unterschieden sich die verschiedenen Studien erheblich in der Zahl der eingeschlossenen Patienten. Zum anderen gibt es große Unterschiede in den Patientencharakteristika und der Art der Datenerhebung. In einigen Studien wurden nur Patienten eingeschlossen, die sich mit nichtdringlichen Problemen vorstellten [5, 14, 21, 32]. In einigen Studien wurden Fragebögen verwendet, oft mit Multiple-Choice-Antwortmöglichkeiten [5, 6, 18, 32]. In einigen Erhebungen wurden persönliche Interviews durchgeführt [8, 11] und in einigen Studien erfolgten Telefoninterviews [15, 30].

Die Prozesse jeder Notaufnahme werden maßgeblich durch den Versorgungsauftrag des Krankenhauses und die demografische Struktur des Patienteneinzugsgebiets bestimmt. In unserem Fall handelt es sich um einen universitären Maximalversorger im Zentrum einer Millionenstadt in unmittelbarer Nähe zu einer großen Bereitschaftspraxis, auch wenn diese organisatorisch nicht an unser Klinikum angeschlossen ist. Umso interessanter wären weiterführende, multizentrische Erhebungen mit Ausweitung auch auf andere Fachbereiche und Einbeziehung von Notaufnahmen beispielsweise in kleineren Städten und ländlichen Bereichen.

## Schlussfolgerung

Aktuell sind die Notaufnahmen von großer Bedeutung für die Versorgung von Patienten, die sich selbständig und ohne Einlieferung durch die Rettungsdienste dort vorstellen. Wie viele dieser Patienten auch in anderen Versorgungsbereichen behandelt werden könnten, ist noch nicht klar. Dazu müssten auch erst ausreichende, qualitativ hochwertige, funktionierende und auch akzeptierte alternative Versorgungsstrukturen, wie mehr Haus- und Facharztpraxen nicht nur, aber vor allem im ländlichen Raum, ärztliche Bereitschaftspraxen und ausreichende Kapazitäten für den kassenärztlichen Bereitschaftsdienst, geschaffen werden.

## Supplementary Information

Abb. Suppl. 1: Fragebogen
