# Supplementary material for: Motives of patients presenting independently at the emergency department—a prospective monocentric observational study
Source: Med Klin Intensivmed Notfmed. 2024 Jan 17;119(7):546–57. [Article in German] doi: 10.1007/s00063-024-01106-2 (PMC11461550; doi:10.1007/s00063-024-01106-2)
Supplement: Supplementary file 1 — Abb. Suppl. 1: Fragebogen [file 63_2024_1106_MOESM1_ESM.docx]

Notaufnahme: Medizinische Klinik IV des Klinikums der Universität München

Leiter: Prof. Dr. med. M. Wörnle

Studie zu den Gründen für die Inanspruchnahme der Notfallambulanz

Uhrzeit bei Betreten der Notaufnahme/Wartebereich _________

Datum _________

Uhrzeit bei Behandlungsbeginn _________

Wie sind Ihre Deutschkenntnisse?

- Muttersprache
- Gut
- Schlecht

Geschlecht

- Weiblich
- Männlich

Alter _________

Haben Sie die deutsche Staatsbürgerschaft?

- Ja
- Nein

Falls Nein, seit wann leben Sie in Deutschland? ________________

Falls Nein, welche Nationalität haben Sie? ________________

Familienstand

- Ledig
- Verheiratet
- Geschieden
- Verwitwet

Welche Krankenversicherung haben Sie?

- Gesetzlich
- Privat
- Keine

Welchen Schulabschluss haben Sie?

- Hauptschulabschluss
- Mittlere Reife/Realschulabschluss
- (Fach-) Abitur
- Hochschulabschluss
- Keinen

Wie ist ihre berufliche Situation?

- Selbstständig/ Freiberufler
- Angestellter
- Beamter
- Schüler/ Student/ Auszubildender
- Arbeitssuchend
- Rentner/Pensionär
- Hausfrau/-mann
- Sonstiges _____________

Beschwerden

Wie dringlich schätzen Sie ihre Beschwerden ein?

- Sehr dringlich
- Dringlich
- Eher nicht dringlich

Dringlichkeit aufgrund *(Mehrfachantworten möglich)*

- ... der Stärke der Beschwerden/Schmerzen
- ... der Zunahme von Beschwerden/Schmerzen
- ... der Angst vor schweren Verläufen/Ursachen

Seit wann haben Sie ihre Beschwerden?

- < 12 h
- 12 - 48 h
- mehrere Tage
- mehrere Wochen
- mehrere Monate

In welchem Bereich haben Sie ihre Beschwerden/Schmerzen? *(Mehrfachantworten möglich)*

- Atmungsorgane
- Kopf/Hals
- Herz/ Kreislauf
- Verdauungssystem/Bauch
- Bewegungsapart/Gelenke
- Rücken
- Haut
- Allgemein/Fieber

Wurden Sie wegen Ihrer heutigen Beschwerden innerhalb des letzten 4 Wochen bereits ärztlich behandelt?

- Ja, beim Hausarzt
- Ja, beim Facharzt
- Ja, im Krankenhaus
- Nein

Gründe für das Aufsuchen der Notaufnahme: *(Mehrfachantworten möglich)*

- Aus eigenem Entschluss
- Empfehlung durch Angehörige
- Empfehlung durch Hausarzt
- Empfehlung durch Facharzt
- Einweisung durch Arzt
- Ich wurde in dieser Klinik schon früher behandelt

Hausärztliche Versorgung

Haben Sie einen Hausarzt?

- Ja

Falls ja, wann haben Sie diesen zuletzt besucht:

- - Innerhalb der letzten 4 Wochen
  - Innerhalb der letzten 3 Monate
  - Innerhalb der letzten 6 Monate
  - Innerhalb des letzten 12 Monate
  - Vor mehr als 12 Monaten
- Nein

Warum haben Sie sich für die Notaufnahme und gegen den Hausarzt/Facharzt entschieden?

*(Mehrfachantworten möglich)*

- Dringlichkeit der Beschwerden
- Keine geöffnete Hausarztpraxis/Facharztpraxis verfügbar
- Notaufnahme hat bessere diagnostische Möglichkeiten
- Notaufnahme hat bessere Behandlungsmöglichkeiten
- Notaufnahme hat täglich 24h geöffnet
- Notaufnahme gut zu erreichen
- Empfehlung/Einweisung durch Hausarzt/ Facharzt

Welche alternativen Anlaufstellen zur Notfallversorgung sind Ihnen bekannt?

*(Mehrfachantworten möglich)*

- Bereitschaftsdienst der kassenärztlichen Vereinigung
- Notfallpraxen
- Rettungsdienst
- Keine
